# Dietary Fibre Intake in Australia. Paper I: Associations with Demographic, Socio-Economic, and Anthropometric Factors

**DOI:** 10.3390/nu10050599

**Published:** 2018-05-11

**Authors:** Flavia Fayet-Moore, Tim Cassettari, Kate Tuck, Andrew McConnell, Peter Petocz

**Affiliations:** 1 Nutrition Research Australia, Level 13 167 Macquarie Street, Sydney 2000, Australia; tim@nraus.com (T.C.); kate@nraus.com (K.T.); andrew@nraus.com (A.M.); 2 Department of Statistics, Macquarie University, Sydney 2109, Australia; peter.petocz@maquarie.edu.au

**Keywords:** dietary fibre, socio-economic, BMI, Australia, National Nutrition Survey, Adequate Intake, Suggested Daily Target

## Abstract

Dietary fibre is important for regular laxation and reduces chronic disease risk. The National Health and Medical Research Council outlines daily fibre intake targets, yet the proportion of the population that meets these targets is unknown. Using the 2011–2012 National Nutrition and Physical Activity Survey, we profiled fibre intake among Australian children and adults. Data from one-day dietary recalls were analysed (*n* = 12,153, ≥2 years) as well as demographic and anthropometric factors. The median fibre intake was 18.2 g (interquartile range [IQR] 13.2–25.0) in children and 20.7 g (IQR 14.3–28.7) in adults. We found that 42.3% (95% CI 40.5–44.1%) of children and 28.2% (95% CI 27.3–29.1%) of adults met the Adequate Intake (AI), and less than 20% of adults met the Suggested Dietary Target (SDT) to reduce the risk of chronic disease. Older children (aged 14–18 years), girls, young adults (19–30 years), males, and those of lower socio-economic status were less likely to meet the AI (*p* < 0.001). Those with a higher energy intake were more likely to meet the AI. Anthropometric measures were not associated with fibre intake or the likelihood of meeting the AI. Fibre is a nutrient of concern in Australian diets, with most children and adults falling short of recommendations. Adolescents, girls, young adults, men, and those of lower socio-economic status were less likely to meet the recommendations and may benefit most from public health interventions.

## 1. Introduction

Dietary fibre is a central component of a healthy diet. Important for regular laxation [1], a large body of observational evidence indicates that a higher fibre intake is associated with reduced chronic disease risk [1,2,3] and longer life [4,5]. In dose-response meta-analyses of prospective cohort studies, additional 7–10 g of fibre per day was associated with a 9% reduction in cardiovascular disease (CVD) [6], a 9% reduction in type 2 diabetes (T2D) [7], a 10% reduction in colorectal cancer [8], and an 11% reduction in all-cause mortality [4]. In Australia, chronic diseases are the leading cause of morbidity and mortality [9], and a recent cost-of-illness analysis reported that if the intake of fibre in Australia increased using cereal fibre, economic savings of AUD$17.8 million–1.6 billion for CVD and AUD$18.2 million–1.7 billion for T2D could be realised in healthcare expenditure and productivity costs [10].

Despite the benefits, the mean intakes of fibre in Australia are below the target intakes. The recommended levels of fibre are outlined by the National Health and Medical Research Council and include the Adequate Intake (AI), based on adequate laxation, and the Suggested Dietary Target (SDT) to reduce chronic disease risk. The AI ranges from 14 to 28 g for children and adolescents (children) aged 1–18 years-old, while, for adults, it is 25 g for women and 30 g for men [11]. The SDT is higher, being 28 g for women and 38 g for men [11]. In the 2011–2012 National Nutrition and Physical Activity Survey (NNPAS), the mean fibre intake per day was 24.8 g for men, 21.1 g for women, and 19.7 g for children, thus lower than the recommended levels for men, women, and most childhood age groups [12], though the proportion of individuals not meeting their fibre targets remains unknown.

Globally, numerous studies have utilised nationally representative data to profile the fibre intakes [13]. The applicability of overseas findings to the Australian context is limited, as fibre intakes and targets are country-specific. In Australia, data on fibre intakes are limited to small cross-sectional studies [14,15] and to particular types of fibre [16], or the data are more than 10 years old [17,18,19]. No analysis of total fibre based on the most recent nationally representative survey data has been conducted.

The aim of this study, therefore, was to determine the fibre intake, the proportion of persons meeting the AI and SDT, and the associations between fibre intake and demographic, socioeconomic, and anthropometric factors in a nationally representative sample of Australian children and adults from the 2011–2012 NNPAS. A more sophisticated understanding of fibre intake in Australia will help to establish the need to address fibre intake inadequacies in at-risk populations.

## 2. Materials and Methods

### 2.1. Survey Methodology

The 2011–2012 NNPAS is a nationally representative survey carried out by the Australian Bureau of Statistics (ABS) and forms part of the 2011–2013 Australian Health Survey. Detailed dietary information and physical activity data were collected for the NNPAS during face-to-face interviews by trained interviewers from the ABS. The data were collected from 12,153 participants aged 2 years and over, 7735 of whom provided dietary data for a second day of recall by telephone interview. Participants were categorised as children (2–18 years, *n* = 2812) or adults (19+ years, *n* = 9341). Interview responses were provided by an adult proxy for children aged 2–8 years, with children aged 6–8 years allowed to assist. An Automated Multiple-Pass Method, developed by the Agricultural Research Service of the United States Department of Agriculture, was used to capture all foods and beverages consumed by the respondents within the 24 h prior to the interview day. The quantity of each food consumed was measured in grams and kilojoules (kJ), and dietary intake data were analysed using the survey specific 2011–2013 Australian Food Composition Database (AUSNUT) developed by Food Standards Australia New Zealand (FSANZ) [20]. To maximise the sample size, only the first day of dietary recall was used for all analyses. The data were weighted to represent the Australian population with weightings provided by the ABS, and the weights were adjusted proportionally so that the total weighted sample size was equal to the total unweighted sample size. The interview components of the survey were conducted under the Census and Statistics Act 1905, and ethics approval was not necessary. Further survey details are available online under the Australian Health Survey: Users’ Guide, 2011–2013 [21].

### 2.2. Fibre Intake and Targets

The total fibre intakes, in grams per day, was determined for all respondents. The children were categorised into the age groups 2–3 years, 4–8 years, 9–13 years, and 14–18 years, and the adults were categorised in the age groups 19–30 years, 31–50 years, 51–70 years, or 71+ years. The AI is defined as an adequate intake based on the appearance or disappearance of gastrointestinal function and adequate laxation and is derived from the median intakes from national dietary surveys in Australia and New Zealand, where laxation problems are considered uncommon [11]. The SDT is defined as an adequate intake to reduce chronic disease risk, on the basis of an inverse association between fibre intake and chronic diseases such as coronary heart disease, and is equivalent to the 90th percentile of intake among adults [11]. There is no SDT for children. The respondents were classified depending on whether they met their age- and sex-specific AI or SDT for fibre.

### 2.3. Demographic and Anthropometric Characteristics

The Socio-Economic Indexes for Areas (SEIFA) [22] was used to define socio-economic status (SES). SEIFA is a product developed by the ABS that ranks areas in Australia into quintiles according to relative socio-economic advantage or disadvantage. The lowest SES quintile was defined as the first quintile, and the highest as the fifth quintile.

Physical measurements including weight, height, and waist circumference were taken for all respondents by trained interviewers during the face-to-face interviews. For children, the body mass index (BMI) z-score was utilised, also known as BMI standard deviation (SD) score, which is a measure of relative weight adjusted for age and sex. The BMI z-score was calculated using the child’s age, sex, height, and weight, and the World Health Organization growth reference standards for 2–4- and 5–9-year-old children [23]. The standard normal distribution was then calculated for all children’s BMI z-scores. This was used to categorise children as: “normal weight” (<85%), “at risk for overweight” (≥85% to <95%), or “overweight” (≥95%). The waist circumference-to-height ratio was calculated for each child. A waist circumference-to-height ratio of <0.5 is associated with a low risk of metabolic complications, whereas a ratio of ≥0.5 is associated with a higher risk [24]. For adults, the body mass index (BMI, kg/m^2^) was calculated using the measured height and weight. The respondents were classified as: underweight (<18.5 kg/m^2^), normal weight (≥18.5 kg/m^2^ to <25 kg/m^2^), overweight (≥25 kg/m^2^ to <30 kg/m^2^), or obese (≥30 kg/m^2^) [25]. Adults were further categorised according to their measured waist circumference and classified on the basis of the World Health Organisation categories for level of risk of metabolic complications: not at risk (<80 cm females, <94 cm males), at increased risk (≥80 cm to <88 cm females, ≥94 cm to <104 cm males), or at substantially increased risk (≥88 cm females, ≥104 cm males) [26].

### 2.4. Underreporting

The basal metabolic rate (BMR) is the amount of energy needed for an individual’s minimum set of body functions necessary for life over a defined period of time. The BMR was measured in kJ per 24 h and calculated using age, sex, and weight (kg) as variables with no adjustment for activity levels. The energy intake-to-basal metabolic rate ratio (EI:BMR) was used to calculate under-reporters, i.e., participants with implausibly low intakes. Participants aged 10 years and over were classified as under-reporters or not under-reporters on the basis of the Goldberg [27] cut-off limit of 0.9 for EI:BMR, which is the lower 95% confidence limit for a single day of data for a single individual, allowing for day-to-day variations in energy intakes and errors in the calculation of EI:BMR.

### 2.5. Statistical Analyses

The statistical package IBM SPSS version 23.0 (IBM Corp., Armonk, NY, USA) was used for all analyses. Because of the large sample sizes, *p*-values < 0.001 were treated as significant. General linear models were used to adjust the mean fibre intake for energy intake, sex, age group, the interaction of sex and age group, SES quintile, BMI (BMI z-score for children), and waist circumference (waist:height ratio for children). Tables of marginal means, standard errors, and 95% confidence intervals were obtained from the analyses. Post hoc pairwise comparisons using the Bonferroni Correction showed pairwise significance between consumer categories. Logistic regression models were used to determine the likelihood of meeting the fibre AI and SDT, adjusted for energy intake, sex, age group, the interaction of sex and age group, SES quintile, BMI (BMI z-score for children), and waist circumference (waist:height ratio for children).

## 3. Results

### Fibre Intakes

In children, the median fibre intake was 18.2 g (IQR 13.2–25.0 g), and less than half of the children (42.3%, 95% CI 40.5–44.1%) met the AI (Table 1). Boys were more likely to meet the AI than girls, and younger children (2–3 years) were more likely to meet the AI than adolescents (14–18 years) (*p* < 0.001).

In a general linear model for fibre intake adjusted for energy intake, demographic, and anthropometric measures, 14–18-year-olds and children in the lowest SES quintile had the lowest fibre intakes (Table 1). There was no difference in fibre intake between boys and girls, and anthropometric measures were not predictors of fibre intake.

In a binary logistic regression, 2–3-year olds were 39 times more likely to meet the AI than adolescents (14–18-year olds) (Table 2). Children with a greater energy intake were more likely to meet the AI. For every additional 1 MJ of energy consumed, there was a 50% increase in the likelihood of meeting the AI. Children of higher SES were also more likely to meet the AI. For each increase in SES quintile, there was a 20% increase in the likelihood of meeting the AI.

In adults, the median fibre intake was 20.7 g (IQR 14.3–28.7 g), and less than a third met the AI, with fewer than one in five meeting the SDT (17.0%, 95% CI 16.2–17.8%) (Table 3). More females (19.9%) met the SDT than males (14.0%), but not the AI. There was no difference in the likelihood of meeting the AI by age group. Young adults (aged 19–30 years) were the least likely to meet the SDT, and those aged 51–70 years were the most likely.

In a general linear model for fibre intake adjusted for energy intake, demographic, and anthropometric measures, young adults (19–30 year olds) had the lowest fibre intake, and those aged 51 years and older had the highest intake (Table 3). Adults in the highest SES quintile had a higher intake than those in the lowest quintile.

In logistic regression models for the likelihood of meeting the AI and SDT, adult females were 2.2 times more likely to meet the AI and 5.3 times more likely to meet the SDT than males (Table 4 and Table 5). Young adults (19–30 years) were the least likely to meet both the AI and the SDT. A greater energy intake was again associated with a greater likelihood of meeting the targets. For every additional 1 MJ of energy consumed there was a 35% increase in the likelihood of meeting the AI and a 33% increase in the likelihood of meeting the SDT. Similar to children, those from higher SES were more likely to meet the targets. For each increase in SES quintile, there was a 9% increase in the likelihood of meeting the AI and a 10% increase the likelihood of meeting the SDT. Waist circumference and BMI were not significant predictors of meeting the AI or the SDT.

Among children aged 10 years and over, 17.2% were under-reporters, and significantly fewer under-reporters met the fibre AI compared to those who did not under-report (6.4% vs. 40.8%, respectively, *p* < 0.001). Among adults, 21.5% were under-reporters, and significantly fewer under-reporters met the fibre AI (5.6% vs. 35.0%, respectively) and fibre SDT (1.8% vs. 21.6%, respectively) compared to adults who did not under-report (*p* < 0.001).

## 4. Discussion

The current analysis of the most recent Australian nationally representative nutrition survey shows that most Australians did not meet fibre targets, with more than one in two children and more than seven in 10 adults not meeting the AI, and more than four in five adults with intakes less than the SDT. The likelihood of meeting the fibre targets varied by age, sex, and socio-economic status, showing that 14–18 year olds were the least likely to meet the AI among children, and 19–30 year-olds and men were the least likely to meet the SDT among adults. The lower socio-economic groups were less likely to meet the recommendations for both children and adults. Fibre intake was not related to anthropometric measures. To our knowledge, this study is the first comprehensive analysis of total fibre intake in the Australian population based on recent nationally representative data.

Our findings demonstrate that fibre is a nutrient of concern in the Australian diet, in line with global nationally representative nutrition survey data. A review of 47 studies in children and 29 studies in adults found that fibre intakes were lower than the recommended intakes for most countries, and the mean intakes were generally around 20 g per day for men and 18–20 g per day for women [13]. Few studies have reported on the proportion of people meeting recommendations. In the U.S., less than 3% of males and 6% of females met the AI in 2003–2006 [28]. In Australia, the unadjusted mean daily fibre intake in the 2007 Australian National Children’s Nutrition and Physical Activity Survey was 20.7 g, and 50% of the children did not meet the AI [19]. In adults, the unadjusted mean fibre intake in the 1995 National Nutrition Survey was 23.1 g, and the median was 21.1 g [29]. Results from the 2008–2009 New Zealand Adult Nutrition Survey reported a mean fibre intake of 20.3 g per day, specifically, 22.8 g for men and 17.9 g for women [30]. These results suggest that fibre intake in Australia is similar to or higher than in other countries and that fibre intakes have remained similar over the last 10–20 years, although direct comparisons between studies are limited by differences in methodology and underreporting [21]. The proportion of persons with a fibre intake below the recommended target should be interpreted cautiously, since no estimated average requirement exists for fibre, and the AI cannot be used to determine inadequacy [11].

The energy-adjusted results showed that adolescents, young adults, and men had the lowest fibre intakes, and this finding confirms that age and sex are important predictors of the likelihood of meeting fibre targets in Australia. The association between fibre intake and age are in line with studies from the U.S., Germany, and Ireland, which found that adolescents consumed less fibre than younger children [31,32], and that younger adults consumed less total fibre than older adults [33], after adjusting for energy intake. Whilst men typically have higher unadjusted fibre intakes than women [13], studies in Spain [34], Ireland [33], Italy [35], and Finland [36] revealed that men had lower intakes of fibre than women when adjusted for energy intake. Other recent analyses from the 2011–2012 NNPAS have reported that, when adjusted for energy intake, these groups generally have lower intakes of core foods and higher intakes of discretionary foods. In all age groups, the intakes of wholegrain were the lowest for adolescents [37]. Among adults, the intakes of fruit and vegetables were lower in males [38], the intakes of discretionary foods and beverages were higher in young adults and males [39], and the diet quality score based on the 2013 Australian Dietary Guidelines was lower for young adults and males [40]. A lower food literacy may contribute to the poorer diets observed for males. Compared to females, males are less likely to have a high level of nutrition knowledge [41,42], to be health-conscious [43,44], and to be involved in meal preparation [45]. Our finding that adolescents are at greater risk of not meeting fibre targets is of concern, as the effects of adolescents’ diet tracks into adulthood [46,47,48]. Their changing neurocognitive maturity and variable social contexts may contribute to the poorer health behaviours of adolescents [49], and the relative cheapness and easy accessibility of less nutritious foods form additional barriers to healthy eating [50]. The less healthy diets of younger adults in comparison to older adults may be related to a lower level of importance attached by this age group to their health [51]. Wide-ranging strategies are needed that address the diet quality, including the intake of fibre, in adolescents, younger adults, and men.

The finding that the socioeconomic status was associated with fibre intake in both children and adults suggests that public health initiatives that address low fibre intakes could prioritise lower SES groups. It is widely established that lower SES groups have poorer dietary habits [52,53,54] also in Australia [40,55,56]. In line with our findings, an association between SES and fibre intake has been reported in children [57,58,59] and adults [60,61,62] in developed nations. Targeting the fibre intake of lower SES groups has the potential to bring about greater health and economic benefits, since lower SES groups have higher levels of health risk factors in Australia [9]. Modelling evidence has estimated that increasing the cereal fibre intake of the lower SES groups of Australian adults results in greater economic savings than implementing the same measure for the higher SES groups [10]. The perceived monetary cost of a healthy diet has been identified as a key factor in the socio-economic differences in dietary intakes [63,64,65]. A recent Australian study found that all SES groups spent more money on unhealthy ‘discretionary’ foods and drinks than on healthy foods and drinks, and that a healthy diet can cost less than an unhealthy diet [66]. Identifying and promoting foods that are good sources of fibre, in line with the Australian Dietary Guidelines, but also affordable and appealing, may help to reduce the observed differences in fibre intake among the SES groups. Differences in dietary knowledge may also contribute to the association between SES and diet [67]. A study in Australian adults found that those in higher socio-economic positions had greater nutrition knowledge than those with a lower SES, and that this factor attenuated most associations between socio-economic position and food purchasing choices [68]. Future research is required to understand the effectiveness of interventions that specifically aim to increase the fibre intake of lower SES populations.

The relationship between fibre and energy intake raises questions about the effects that recommendations to increase fibre intake could have on increasing total energy intake. This concern is supported by intervention evidence, with a large randomised controlled trial showing that an increased total fibre intake achieved by increasing whole grain food consumption also increased the total energy intake [69]. Whilst a higher fibre intake was associated with a higher energy intake in our study, a higher fibre diet is generally associated with a lower-energy-density diet (energy density is measured in kJ per unit weight of food) [70], and several fibre types have been shown to reduce subsequent food or energy intake in systematic reviews of controlled trials [71,72]. Thus, increasing fibre intake can also reduce total energy intake, if total food intake is controlled. In our study, we did not analyse the daily food intake by weight and so did not investigate this phenomenon. The recommendations to increase fibre may need to focus on the substitution of low-fibre foods with high-fibre foods, such as wholegrains versus refined grains, rather than increasing the intake of high-fibre foods only. Future research on the dietary patterns of high- and low-fibre consumers is required to help inform effective dietary recommendations.

We found nil or inverse associations between fibre intake and anthropometric measures. There is considerable evidence that a greater fibre intake is associated with a lower obesity risk in adults [1,72,73,74,75] and children [73,76], including consistent evidence from nationally representative data in the U.S. [75] and a systematic literature review of randomised controlled trials [72], although not all studies report an inverse association. In Poland, two cross-sectional studies reported no association between fibre intake and BMI in adolescents or young adults [57,77]. In a prospective study of German adolescents, fibre intake was not associated with body fat percentage or BMI [78]. In a study of Latino children, fibre was also not associated with adiposity [79]. Further differences between studies could be due to differences in the fibre type. In a longitudinal study in Europe, a higher fibre intake from cereal, but not fruit or vegetable, was associated with lower body weight [80]. A review of intervention evidence also reported different effects depending on the physiochemical properties of the fibre, with stronger effects for more vicious, more soluble, and more fermentable fibres [72]. The associations may also be influenced by under- and over-reporting. A recent study reported that fibre intake among Spanish adults was lower among overweight and obese, but when under- and over-reporters were excluded, these differences were no longer observed [34]. High-fibre foods contain other nutrients and non-nutrients. Whole grains are rich in magnesium, B vitamins, zinc, phenolic compounds, and phytoestrogens [6,81], while fruits and vegetables contain antioxidant vitamins, folate, flavonoids, phenols, and plant sterols [82]. It is likely that these other components found in high-fibre foods may contribute to the beneficial effects of fibre on the reduction of chronic disease risk reported in epidemiological studies [82,83,84]. Further, our data are cross-sectional and may be subject to confounding or reverse causation. The nature of the association between fibre intake and anthropometric measures remains unclear and requires further research.

The strengths of our study include the use of a large nationally representative sample of children and adults, making our results generalisable to the Australian population. Further, our analysis is adjusted for energy intake wherever relevant, a parameter that was strongly associated with total fibre intake. Despite the lack of an estimated average requirement for fibre, we have included analyses using the Suggested Dietary Target for adults. The limitations of this study should also be considered. As with other nutrition surveys, fibre intake from the 2011–2012 NNPAS may have been affected by underreporting [21]. We found that under-reporters were significantly less likely to meet fibre targets. This may be explained in part by a proportion of low-fibre consumers actively dieting and purposely reducing their total energy intake, which was related to fibre intake and not specifically related to under-reporting as predicted by the EI:BMR ratio. In general, fibre intake data may underestimate the actual fibre intakes, since food composition databases may not capture all fibre types, such as resistant starch [11]. We used one day of dietary recall to categorise participants and estimate means in order to increase the sample size, which may not reflect the usual intakes, although there is a small variation between usual intake and population means. Finally, these data are cross-sectional, precluding any causal relationships.

## 5. Conclusions

The majority of Australian children and adults did not meet fibre targets. The greatest fibre shortfalls were observed among adolescents, younger adults, men, those of lower socio-economic status, and those with lower daily energy intakes. Given the costs of chronic disease in Australia and the role that fibre plays in preventing these diseases, our findings strengthen the need for interventions and initiatives that aim to increase fibre intakes in line with the Australian Dietary Guidelines. Further research should focus on the food sources of fibres to help inform dietary recommendations for such initiatives.

## Figures and Tables

**Table 1:** Total daily fibre intake and the proportion of children aged 2–18 years that met the fibre AI by age, sex, SES, and anthropometric group.

		Fibre Intake (grams)		Proportion that Met the Fibre AI *
		Adjusted ^†^ Mean ± SE	Median [IQR]	% [95% CI]
All Children	2–18 Years	19.9 ± 0.2	18.2 [13.2, 25.0]	42.3 [40.5, 44.1]
All children	2–3 years	20.1 ± 0.6 ^a,b^	15.3 [10.5, 20.0]	58.4 [53.1, 63.7]
	4–8 years	20.8 ± 0.3 ^a^	17.8 [13.2, 23.0]	48.0 [44.6, 51.5]
	9–13 years	19.8 ± 0.3 ^a,b^	19.5 [14.1, 26.4]	42.3 [39.1, 45.6]
	14–18 years	18.8 ± 0.3 ^b^	18.9 [13.3, 26.0]	29.4 [26.2, 32.7]
*p* value		<0.001		<0.001
All children	Boys	20.0 ± 0.3	19.4 [14.0, 26.5]	44.1 [41.5, 46.7]
	Girls	19.8 ± 0.3	17.0 [12.4, 22.8]	40.5 [37.9, 43.1]
*p* value		0.091		<0.001
Boys	2–3 years	20.6 ± 0.7	16.6 [9.8, 22.9]	62.0 [54.8, 69.3]
	4–8 years	20.7 ± 0.5	19.4 [13.4, 24.5]	54.9 [50.1, 59.6]
	9–13 years	20.4 ± 0.4	21.1 [15.3, 28.3]	41.5 [37.0, 46.1]
	14–18 years	18.4 ± 0.5	19.8 [13.6, 28.4]	27.6 [23.1, 32.0]
Girls	2–3 years	19.7 ± 0.7	15.1 [10.9, 18.7]	54.5 [46.7, 62.2]
	4–8 years	20.8 ± 0.5	16.3 [11.7, 21.6]	40.7 [35.8, 45.6]
	9–13 years	19.3 ± 0.4	18.1 [12.9, 25.3]	43.2 [38.6, 47.8]
	14–18 years	19.3 ± 0.5	17.9 [12.9, 23.9]	31.3 [26.7, 36.0]
*p* value		0.091		<0.001
zBMI group ^‡^	Normal weight	19.6 ± 0.2	18.8 [13.6, 25.1]	41.3 [38.9, 43.8]
	At risk of overweight	20.0 ± 0.5	16.7 [12.3, 23.6]	41.1 [35.5, 46.7]
	Overweight	20.0 ± 0.4	18.1 [12.4, 25.3]	45.4 [40.8, 50.1]
*p* value		0.697		0.286
Risk of metabolic complications ^§^	Not at risk	20.2 ± 0.3	19.0 [14.0, 25.7]	40.9 [38.4, 43.3]
	Increased risk	19.6 ± 0.3	16.8 [12.0, 23.3]	44.6 [41.0, 48.2]
*p* value		0.170		0.049
SES ^||^ quintile	Lowest	18.8 ± 0.4 ^a^	15.9 [11.4, 23.1]	33.3 [29.1, 37.5]
	2nd	19.6 ± 0.4 ^a,b^	18.2 [13.3, 24.0]	41.3 [37.1, 45.5]
	3rd	19.4 ± 0.4 ^a,b^	17.0 [12.1, 23.8]	38.9 [35.0, 42.8]
	4th	20.2 ± 0.4 ^a,b^	18.6 [13.7, 25.6]	44.3 [40.0, 48.6]
	Highest	21.3 ± 0.4 ^b^	20.3 [14.5, 26.4]	51.1 [47.3, 54.8]
*p value*		<0.001		<0.001

Abbreviations: AI, adequate intake; SES, socio-economic status; SD, standard deviation; SE, standard error; IQR, inter-quartile range; CI, confidence interval; zBMI, body mass index-for-age-z-score; * AI is 14 g for boys and girls 2–3 years old, 18 g for boys and girls 4–8 years old, 24 g for boys and 20 g for girls aged 9–13 years, 28 g for boys and 22 g for girls aged 14–18 years [11]. ^†^ Adjusted for sex, age group, their interaction, SES quintile, zBMI group, waist:height ratio group, and energy intake. Age group, SES quintile, and energy intake were significant, R-squared = 0.387. ^a,b^ A different superscript denotes a significant difference between groups (*p* < 0.001). ^‡^ Calculated using the standard normal distribution of BMI z-scores:normal weight (<85%), at risk for overweight (≥85% to <95%), overweight (≥95%) [23]. ^§^ In children, a waist circumference-to-height ratio of <0.5 is associated with a low risk of metabolic complications from obesity, whereas a ratio of >0.5 is associated with a higher risk [24]. Therefore, a waist circumference-to-height ratio of 0.5 was used as a cut-off for waist circumference and risk of metabolic complications. ^||^ Based on Socio-Economic Indexes for Areas (SEIFA) [22], a product developed by the Australian Bureau of Statistics (ABS) that ranks areas in Australia according to their relative socio-economic advantage.

**Table 2:** Binary logistic regression analysis of predictors of meeting fibre AI * in children aged 2–18 years.

Predictor	Β (SE)	OR [95% CI]	*p* Value
Energy (MJ)	0.403 (0.022)	1.496 [1.432, 1.562]	<0.001
Age group			<0.001
4–8 y	−0.958 (0.244)	0.384 [0.238, 0.619]	<0.001
9–13 y	−2.393 (0.259)	0.091 [0.055, 0.152]	<0.001
14–18 y	−3.666 (0.293)	0.026 [0.014, 0.045]	<0.001
(Ref = 2–3 y)			
Sex	−0.277 (0.279)	0.758 [0.439, 1.309]	0.320
(Ref = M)			
Age group * sex			<0.001
4–8 y * M	0.129 (0.331)	1.137 [0.594, 2.177]	0.698
9–13 y * M	0.936 (0.326)	2.550 [1.345, 4.835]	0.004
14–18 y * M	1.647 (0.353)	5.191 [2.600, 10.361]	<0.001
(Ref = 2–3 y * M)			
zBMI	0.129 (0.057)	1.138 [1.016, 1.273]	0.025
Waist: height ratio	−0.676 (1.212)	0.508 [0.047, 5.474]	0.577
SES ^†^	0.180 (0.035)	1.198 [1.118, 1.282]	<0.001
Constant	−2.167 (0.692)	0.115	0.002

AI, adequate intake; Β, regression coefficient; SE, standard error; OR, odds ratio; CI, confidence interval; MJ, Megajoules; y, years; M, males; zBMI, body mass index-for-age-z-score; SES, socio-economic status. * AI is 14 g for boys and girls aged 2–3 years, 18 g for boys and girls aged 4–8 years, 24 g for boys and 20 g for girls aged 9–13 years, 28 g for boys and 22 g for girls aged 14–18 years [11]. ^†^ Based on Socio-Economic Indexes for Areas (SEIFA) [22], a product developed by the ABS that ranks areas in Australia according to their relative socio-economic advantage.

**Table 3:** Total daily fibre intake and the proportion of adults aged 19 years and over that met the fibre AI and SDT by age, sex, SES, and anthropometric measures.

		Fibre Intake (grams)		Proportion that Met the Fibre AI *	Proportion that Met the Fibre SDT ^†^
		Adjusted ^‡^ Mean ± SE	Median [IQR]	% [95% CI]	% [95% CI]
**All Adults**	**19+ Years**	**23.8 ± 0.3**	**20.7 [14.3, 28.7]**	**28.2 [27.3, 29.1]**	**17.0 [16.2, 17.8]**
All adults	19–30 years	21.1 ± 0.3 ^a^	20.1 [13.7, 28.3]	25.8 [24.0, 27.7]	14.9 [13.4, 16.4]
	31–50 years	22.7 ± 0.3 ^b^	20.7 [14.5, 28.4]	27.6 [26.1, 29.1]	16.3 [15.0, 17.5]
	51–70 years	24.8 ± 0.3 ^c^	21.1 [14.9, 29.9]	30.7 [29.0, 32.5]	19.2 [17.8, 20.7]
	71+ years	26.5 ± 0.4 ^c^	20.5 [14.4, 28.3]	28.7 [25.9, 31.5]	18.1 [15.7, 20.5]
*p* value		<0.001		0.002	<0.001
All adults	Males	23.6 ± 0.3	22.4 [15.7, 31.5]	28.5 [27.2, 29.8]	14.0 [13.0, 15.0]
	Females	24.0 ± 0.3	19.1 [13.3, 26.1]	27.9 [26.6, 29.2]	19.9 [18.8, 21.1]
*p* value		0.196		0.255	<0.001
Males	19–30 years	20.2 ± 0.4	22.8 [15.5, 30.8]	26.4 [23.8, 29.0]	11.3 [9.5, 13.2]
	31–50 years	22.6 ± 0.4	22.4 [16.2, 31.8]	28.7 [26.6, 30.8]	14.0 [12.4, 15.7]
	51–70 years	24.4 ± 0.4	22.1 [15.2, 32.1]	30.2 [27.7, 32.6]	15.7 [13.7, 17.6]
	71+ years	27.2 ± 0.6	22.4 [16.2, 32.1]	28.2 [24.0, 32.3]	15.7 [12.3, 19.0]
Females	19–30 years	22.1 ± 0.4	18.0 [12.2, 25.1]	25.3 [22.6, 27.9]	18.6 [16.3, 20.9]
	31–50 years	22.8 ± 0.4	19.0 [13.2, 25.5]	26.5 [24.4, 28.6]	18.5 [16.7, 20.3]
	51–70 years	25.2 ± 0.4	20.1 [14.3, 27.4]	31.2 [28.8, 33.7]	22.7 [20.5, 24.9]
	71+ years	25.9 ± 0.6	19.4 [13.5, 26.2]	29.1 [25.3, 32.9]	20.1 [16.8, 23.5]
*p* value		0.002		0.012	<0.001
BMI group ^§^	Underweight	24.5 ± 0.9	18.1 [13.1, 28.9]	29.4 [22.0, 36.9]	18.9 [12.5, 25.3]
	Normal weight	24.3 ± 0.3	21.8 [15.0, 30.6]	32.6 [30.9, 34.4]	21.7 [20.2, 23.2]
	Overweight	23.8 ± 0.2	21.6 [15.1, 30.0]	30.0 [28.3, 31.6]	16.9 [15.5, 18.3]
	Obese	22.6 ± 0.3	19.2 [13.5, 26.4]	21.9 [20.1, 23.7]	12.0 [10.6, 13.4]
*p* value		0.002		<0.001	<0.001
Risk of metabolic complications ^||^	Not at risk	24.4 ± 0.3	22.0 [15.2, 31.3]	32.3 [30.6, 33.9]	19.5 [18.1, 21.0]
	Increased risk	23.5 ± 0.4	21.4 [14.7, 29.4]	28.8 [26.7, 30.9]	17.0 [15.3, 18.8]
	Substantially increased risk	23.5 ± 0.4	19.8 [14.1, 27.4]	25.6 [24.1, 27.1]	15.6 [14.4, 16.9]
*p* value		0.031		<0.001	<0.001
SES ^¶^ quintile	Lowest	23.1 ± 0.4 ^a^	19.0 [13.2, 27.4]	24.2 [22.2, 26.3]	15.3 [13.6, 17.0]
	2nd	23.5 ± 0.3 ^a,b^	20.2 [14.1, 27.7]	25.7 [23.7, 27.6]	14.0 [12.4, 15.5]
	3rd	23.7 ± 0.3 ^a,b^	20.8 [14.4, 28.8]	29.3 [27.3, 31.3]	16.7 [15.0, 18.4]
	4th	24.0 ± 0.4 ^a,b^	21.5 [15.2, 28.9]	28.2 [26.1, 30.3]	17.9 [16.1, 19.7]
	Highest	24.9 ± 0.3 ^b^	21.9 [15.1, 30.4]	32.7 [30.7, 34.8]	20.6 [18.9, 22.4]
*p* value		<0.001		<0.001	<0.001

Abbreviations: AI, adequate intake; SDT, suggested dietary target; SD, standard deviation; SE, standard error; IQR, inter-quartile range; CI, confidence interval; SES, socio-economic status; BMI, body mass index. * AI is 30 g for males, 25 g for females [11]. ^†^ SDT is 38 g for males, 28 g for females [11]. ^‡^ Adjusted for sex, age, their interaction, SES quintile, BMI, waist circumference, and energy intake. Age, SES quintile and energy intake were significant, R-squared = 0.293. ^a,b,c^ A different superscript denotes a significant difference between groups (*p* < 0.001). ^§^ Based on BMI: underweight (<18.5), normal weight (≥18.5, <25.0), overweight (≥25.0, <30.0), obese (≥30.0) [25]. ^||^ Based on World Health Organization cut-offs for waist circumference: not at risk of metabolic complications (females: <80 cm; males: <94 cm); increased risk of metabolic complications (females: ≥80 cm, <88 cm; males: ≥94 cm, <102 cm); substantially increased risk of metabolic complications (females: >88 cm; males: >102 cm) [26]. ^¶^ Based on Socio-Economic Indexes for Areas (SEIFA) [22], a product developed by the ABS that ranks areas in Australia according to their relative socio-economic advantage.

**Table 4:** Binary logistic regression analysis of predictors of meeting fibre AI^*^ among adults aged 19 years and over.

Predictor	Β (SE)	OR [95% CI]	*p* Value
Energy (MJ)	0.296 (0.009)	1.345 [1.321, 1.370]	<0.001
Age group			<0.001
31–50 y	0.467 (0.105)	1.596 [1.300, 1.959]	<0.001
51–70 y	0.854 (0.113)	2.348 [1.880, 2.932]	<0.001
71+ y	1.315 (0.154)	3.724 [2.751, 5.042]	<0.001
(Ref = 19–30 y)			
Sex	0.772 (0.124)	2.164 [1.698, 2.758]	<0.001
(Ref = M)			
Age group * sex			0.168
31–50 y * M	−0.246 (0.149)	0.782 [0.584, 1.047]	0.098
51–70 y * M	−0.157 (0.154)	0.855 [0.632, 1.156]	0.308
71+ y * M	−0.423 (0.204)	0.655 [0.439, 0.976]	0.038
(Ref = 19–30 y * M)			
BMI (kg/m^2^)	−0.004 (0.011)	0.996 [0.975, 1.017]	0.724
Waist circumference (cm)	−0.011 (0.004)	0.989 [0.980, 0.997]	0.009
SES ^†^	0.086 (0.020)	1.090 [1.049, 1.133]	<0.001
Constant	−3.643 (0.254)	0.026	<0.001

AI, adequate intake; Β, regression coefficient; SE, standard error; OR, odds ratio; CI, confidence interval; MJ, Megajoules; y, years; M, males; BMI, body mass index; kg, kilograms; m, metres; cm, centimetres; SES, socio-economic status. * AI is 30 g for males, 25 g for females [11]. ^†^ Based on Socio-Economic Indexes for Areas (SEIFA) [22], a product developed by the ABS that ranks areas in Australia according to their relative socio-economic advantage.

**Table 5:** Binary logistic regression analysis of predictors of meeting fibre SDT^*^ among adults aged 19 years and over.

Predictor	Β (SE)	OR [95% CI]	*p* Value
Energy (MJ)	0.283 (0.010)	1.327 [1.301, 1.354]	<0.001
Age group			<0.001
31–50 y	0.629 (0.138)	1.875 [1.430, 2.460]	<0.001
51–70 y	1.122 (0.147)	3.072 [2.302, 4.099]	<0.001
71+ y	1.721 (0.192)	5.589 [3.834, 8.147]	<0.001
(Ref = 19–30 y)			
Sex	1.673 (0.154)	5.329 [3.941, 7.206]	<0.001
(Ref = M)			
Age group * sex			<0.001
31–50 y * M	−0.498 (0.180)	0.608 [0.427, 0.865]	0.006
51–70 y * M	−0.547 (0.185)	0.579 [0.402, 0.832]	0.003
71+ y * M	−0.980 (0.241)	0.375 [0.234, 0.602]	<0.001
(Ref = 19–30 y * M)			
BMI (kg/m^2^)	−0.028 (0.013)	0.972 [0.948, 0.997]	0.028
Waist circumference (cm)	−0.005 (0.005)	0.995 [0.985, 1.005]	0.332
SES ^†^	0.092 (0.023)	1.096 [1.047, 1.147]	<0.001
Constant	−4.753 (0.308)	0.009	<0.001

SDT, suggested dietary target; Β, regression coefficient; SE, standard error; OR, odds ratio; CI, confidence interval; MJ, Megajoules; y, years; M, males; BMI, body mass index; kg, kilograms; m, metres; cm, centimetres; SES, socio-economic status. * SDT is 38 g for males, 28 g for females [11]. ^†^ Based on Socio-Economic Indexes for Areas (SEIFA) [22], a product developed by the ABS that ranks areas in Australia according to their relative socio-economic advantage.
